# Advancements in epilepsy classification: Current trends and future directions

**DOI:** 10.1016/j.mex.2025.103257

**Published:** 2025-03-07

**Authors:** Rajani Rai B, Karunakara Rai B, Mamatha A S, Kavitha Sooda

**Affiliations:** a Dept.of ECE, Vivekananda College of Engineering and Technology, Puttur, D.K., India; b Dept. of ECE, Nitte Meenakshi Institute of Technology, Bangalore, India; c Dept. of ECE, NITTE (Deemed to be University, NMAM Institute of Technology, Nitte, India; d Dept. of CSE, BMS College of Engineering, Bangalore, India

**Keywords:** Artifacts, Classifiers, EEG, Feature extraction, Focal, Non-focal, Hybrid approaches, Multimodal fusion, Seizure prediction

## Abstract

This paper presents a comprehensive survey on categorizing focal and non-focal epilepsy using Electroencephalogram (EEG) signals. It emphasizes how recent advances in machine learning and deep learning methodologies assists in overcoming the existing challenges in classification. The paper synthesizes cutting-edge techniques with the focus on the application of hybrid models that combine traditional signal processing techniques with machine learning algorithms. By highlighting key breakthroughs in the field, the paper aims to propose novel directions for improving classification precision. Furthermore, the paper delves into the challenges faced by current methods and the possible solutions. The paper concludes with the discussion on potential future research directions, especially in areas of multimodal data integration and real-time seizure prediction, and emphasizes the potential for AI-driven personalized epilepsy treatment techniques.

## Review Highlights

• Classification of focal and non-focal EEG signals.
• Discussion of the different methods of feature extraction., like time domain,frequency domain and wavelets.
• Discussion of classifiers and deep learning methods.
• Classifier relevance in clinical analysis is explained.

## Graphical abstract

**Figure:**
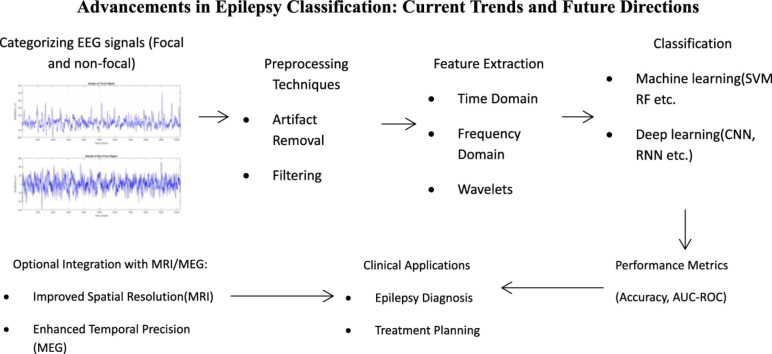

## Specifications table

This table provides general information on the methodology you reviewed.

**Table:** 

Subject area:	Computer Science
More specific subject area:	Signal Processing, Machine learning, deep learning
Name of the reviewed methodology:	Preprocessing, Feature Extraction, Classification
Keywords:	EEG, focal, non-focal, feature extraction
Resource availability:	NA
Review question:	1. What are the key challenges discussed in the paper regarding focal and non-focal epilepsy classification using EEG signals? 2. Does the paper provide an overview of the existing EEG database resources for classifying focal and non-focal epilepsy? 3. Has the paper addressed the necessity and discuss the various types of preprocessing techniques? 4. Has the survey presented the detailed qualitative information about various feature extraction techniques? 5. Does the paper give clear descriptions of classifier types within the framework of EEG-based epilepsy detection? 6. Does this paper effectively demonstrate how multimodal analysis contributes to epilepsy diagnosis?

## Background

**Fig. 1:**
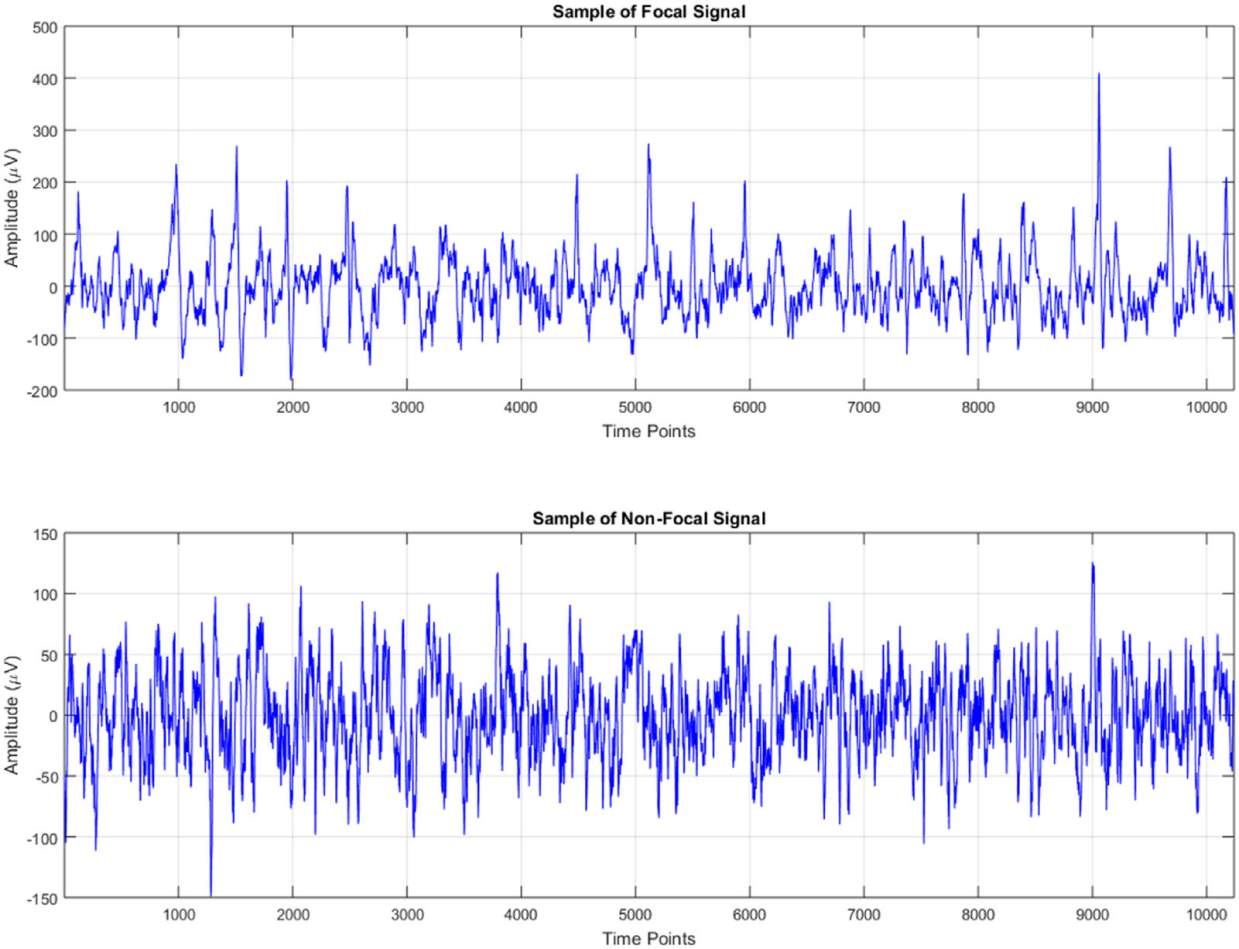
Sample of Focal and Non-Focal EEG signal.

Epilepsy is a chronic neurological condition characterized by repeated seizures, impacting millions globally. With approximately 50 million individuals impacted globally, it represents a significant public health concern [1]. The episodes are generally categorized as focal epilepsy and non-focal epilepsy. Focal epilepsy originates in a specific region of the brain, while non-focal involves more widespread brain activity [2]. Fig. 1 shows a sample of focal and non-focal signal. Distinguishing between these two types is crucial, as it directly influences treatment decisions, such as the choice of antiepileptic drugs, surgical options and other therapeutic interventions. Accurate diagnosis plays a vital role in improving treatment plans and enables a more personalized approach to patient care [3]. Electroencephalography (EEG) is a diagnostic tool commonly used to track brain activity in real-time by positioning electrodes over the scalp [4]. With its high temporal resolution, typically EEG is perfect for tracking the brain's activity as seizures occur. This capability is crucial since seizures often occur suddenly and unpredictably, so it's critical to detect them quickly and accurately for timely intervention. Traditional EEG analysis often depends on clinicians manually reviewing waveforms to classify seizures. This approach is slow, can be prone to human error and is subjective [5].

Consequently, automated machine approaches are developed to make seizure classification faster, more accurate and consistent especially for large datasets where manual analysis is not feasible [6]. Deep learning (DL) has emerged as a powerful approach for EEG analysis, capable of extracting key features from raw EEG data without manual intervention[7]. It captures complex patterns in EEG signals without relying on manual feature extraction. These models have proven to be highly effective, improving classification accuracy across different patient groups [8].

Despite significant progress in this area, accurate categorization of focal and non-focal epilepsy still remains a major challenge. Major factors that contribute to this complexity are:

• **Data Quality**: EEG signals are highly sensitive to noise and artifacts from both internal sources and external sources. Proper preprocessing is critical for mitigating these effects, but it is often computationally expensive and can result in data loss[9].
• **Signal Complexity**: Seizures, especially non-focal ones, exhibit highly non-stationary behavior, making it difficult to extract relevant features for accurate classification. The seizure dynamics may change over time, further complicating classification.
• **Real-time Processing**: In clinical practice, **real-time seizure detection** is essential for timely intervention. Current models, while promising, often face challenges in achieving the **low-latency** and **high-throughput** needed for real-time applications. Most models still rely on **offline** analysis, which is not feasible for continuous monitoring.
• **Class Imbalance**: There is often a significant imbalance in datasets, with more instances of non-focal seizures, leading to model biases. This imbalance makes it challenging for models to effectively learn the subtle features of focal seizures, which are less frequent but clinically more critical.
• **Generalization**: Models trained on specific datasets may not generalize well to new patient populations, due to **inter-patient variability**. This reduces the practicality of existing methods in diverse healthcare settings [10].

**Fig. 2:**
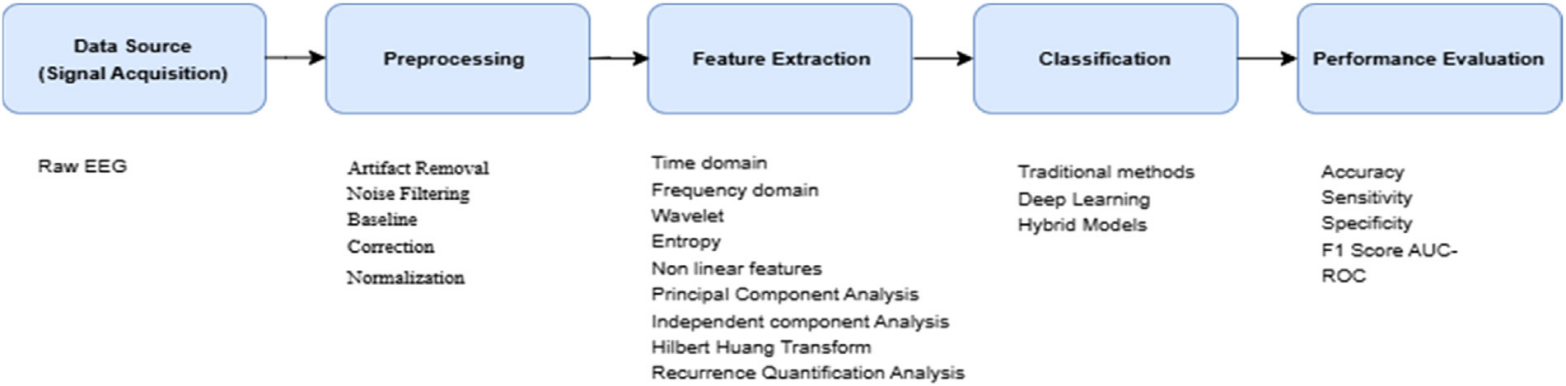
Steps involved in the classification process of EEG signal.

These challenges demand more robust models, particularly through the integration of deep learning techniques, multimodal data and real-time processing systems. The primary objective of this paper is to review the state-of-the-art classification techniques using traditional signal processing methods and Machine Learning (ML) approaches including deep learning. Furthermore, the potential of integrating multimodal data, such as EEG with MRI and genetic information, will be explored to provide holistic understanding of epilepsy. Fig. 2 depicts the basic steps involved in classification process.

## Method details

### Data sources

Access to various, high-quality datasets is critical for accurately diagnosing focal and non-focal epilepsy using EEG signals. These datasets reflect the complexity and variability of epilepsy, with challenges such as data imbalance, variability in patient characteristics, and differences in seizure types.

Table 1 provides an overview of widely used databases for epilepsy classification. The Bonn EEG dataset is composed of five subsets. It is labelled as A, B, C, D and E containing 500 EEG recordings from both healthy and epileptic individuals. It includes data from surface EEG (healthy states) and intracranial EEG (seizure-free and seizure states) with a sampling rate of 173.61 Hz, filtered between 0.53 Hz and 40 Hz. This dataset is widely used for seizure detection and epilepsy research [11]. CHB-MIT dataset contains 844 h of continuous EEG data from 22 paediatric patients (aged 1.5 to 19 years) with 200 recorded seizures. It includes multi-channel EEG recordings (24–27 channels) with sampling frequency of 256 Hz containing separate files for seizure and non-seizure episodes [12]. Epileptic EEG Dataset-Mendeley repository EEG dataset, acquired from six focal epilepsy patients during pre-surgical evaluation, comprises ictal, pre-ictal, inter-ictal and seizure onset signals. Recorded at a 500 Hz sampling rate and stored in EDF format, the data is meticulously labeled for complex partial and video-detected seizures, with all signals band-pass filtered between 1 and 70 Hz to eliminate 50 Hz noise [13]. The Bern-Barcelona EEG dataset contains recordings from five patients with drug-resistant temporal lobe epilepsy. It includes signals recorded at 512 or 1024 Hz, depending on the number of channels, with each recording lasting 20 s and containing 10,240 samples [14].

**Table 1:** Summary of major public EEG datasets used in epilepsy research.

Dataset	Number of Patients	No of channels	Recording Type	Features
Bonn EEG Dataset	5	100 single-channel	Surface and Intracranial EEG	The dataset contains 5 sets (A, B, C, D, E), each representing different conditions or seizure states of patients
CHB-MIT Epilepsy Database	22	24–27	Scalp electrodes	Data from pediatric patients with intractable seizures.
Epileptic EEG Dataset-Mendeley repository	6	21	Scalp electrodes	Complex partial, electrographic and video-detected seizures
Bern Barcelona	5	64	Intracranial EEG recordings	Focal and non-focal data

The categorization of focal and non-focal epilepsy relies heavily on the availability of diverse and high-quality datasets. These datasets reflect the complexity and variability of epilepsy, with challenges such as data imbalance, variability in patient characteristics and differences in seizure types. Furthermore, the scarcity of labeled data for certain seizure types adds another layer of difficulty in developing effective models.

### Preprocessing techniques

Prior to feature extraction, preprocessing is critical for improving the quality and reliability of EEG signals. Raw EEG signals are often affected by noise and artifacts such as muscle movements, eye blinks, or electrical interference. Therefore, appropriate preprocessing techniques are vital for improving the Signal-to-Noise Ratio (SNR) ratio and ensuring success of subsequent classification models. Table 2 provides a summary of preprocessing methods commonly employed in EEG analysis. The key methods and techniques for EEG preprocessing include:

i). Artifact Removal
  Artifact removal is essential for ensuring that EEG signals represent true neural activity rather than being affected by external or physiological noise. Several methods are employed for artifact removal:
  • Independent Component Analysis (ICA): This technique decomposes the EEG signal into independent components, isolating artifacts such as eye blinks, muscle activity, and heartbeats, which can then be removed [15].
  • Regression-Based Methods: These methods use regression models to estimate and subtract artifact-related components, typically those associated with eye movements or muscle activity [16].
  • Wavelet Transform: This approach decomposes EEG signals into time-frequency components, allowing the selective removal of artifacts by filtering in both temporal and spectral domains [17].
ii). Noise Filtering
  Noise filtering is a necessary procedure for improving EEG signal quality by eliminating unwanted frequency components. Various filtering techniques are used:
  • Band-Pass Filtering: This method allows signals within a specific frequency range (e.g., 0.5–70 Hz) to pass through while filtering out high- and low-frequency noise. Commonly used in EEG preprocessing, it retains the most relevant brainwave frequencies while discarding unwanted components
  • Notch Filtering: This filter removes narrow-band interference, such as powerline noise at 50 Hz or 60 Hz, that often contaminates EEG data [18]
  • Adaptive Filtering: This technique adjusts the filter dynamically and adapts to signal variations, enabling real-time noise reduction [19].
iii). Baseline correction
  Baseline correction is applied to eliminate low-frequency drifts and offsets from signals, aligning the data to a consistent baseline period [20]. Common methods include:
  • Mean Subtraction: This method subtracts the average value of signal over a reference period to remove any slow drifts, ensuring that the observed changes are due to neural activity.
  • Linear Detrending: Linear detrending removes any linear trends in the signal by subtracting a best-fit linear line calculated over the baseline period [21] .
  • Wavelet-Based Baseline Correction: This method combines wavelet transform with baseline correction, effectively removing drifts while preserving the signal's key frequency components [22]
iv). Normalization
  Normalization standardizes EEG signals across channels and subjects, improving the comparability and consistency of data [23]. Key methods include:
  • Z-score Normalization: This method standardizes the data by removing mean and scaling it using standard deviation, ensuring that all channels have a similar range and are comparable across subjects.
  • Min-Max Scaling: This approach scales the EEG signal to a fixed range, typically [0, 1], which is particularly useful when the data is processed for machine learning applications.
  • RMS Normalization: Root mean square (RMS) normalization scales the data based on the signal's energy, helping to correct amplitude variations across channels.

**Table 2:** Summary of preprocessing methods used in EEG signal processing.

Preprocessing Method	Description	Challenges	Use Case
Artifact Removal	Techniques such as Independent Component Analysis (ICA) remove artifacts like eye blinks.	Requires careful parameter tuning to avoid losing valid data.	Effective in reducing noise from muscle or eye artifacts.
Noise Filtering	Use of band-pass filters (e.g., 1–50 Hz) to remove high-frequency and low-frequency noise.	Sensitive to filter parameters, may distort signals if not tuned properly.	Essential for isolating seizure-related frequencies from general brain activity.
Baseline correction	Removes low-frequency drifts or offsets in EEG signals	Selecting an appropriate baseline period and addressing non-stationary drifts or physiological variability.	Removes drifts, ensuring focus on neural activity patterns.
Normalization	Scales signal amplitudes to standard ranges to reduce variability.	Loss of small but important variations.	Ensures consistency across different datasets and subjects.

Preprocessing electroencephalographic (EEG) signals is essential as it improves data quality and model accuracy. These methods play a vital role in preprocessing EEG signals, making sure that the data used in subsequent analyses is clean, standardized, and reflective of true neural activity.

### Feature extraction methods

Raw EEG signals are transformed into meaningful representations that machine learning models can use for classification. This process can be divided into traditional and advanced methods. The features extracted from EEG signals plays a crucial role in classifying focal and non-focal epilepsy. Traditional methods typically rely on manual feature selection, which can limit the ability to capture the full complexity of seizure dynamics. Table 3 summarizes the various types of features utilized in EEG signal analysis.

**Table 3:** Summary of Feature Types for EEG Signal Analysis.

Feature Type	Description	Methods	Challenges
Time-domain Features	Characterize the central tendency and variability of the signal.	Mean, variance, skewness, kurtosis	May miss transient or subtle features of seizures.
Frequency-domain Features	Identify abnormalities in the frequency components of the EEG signal.	Fourier Transform (FT), Power Spectral Density (PSD)	Struggles with non-stationary signals like non-focal seizures.
Wavelet-based Features	Capture time-frequency characteristics of EEG signals.	Continuous Wavelet Transform (CWT)	Computationally intensive.
Entropy-based Features	Measure the unpredictability or complexity of the EEG signal.	Approximate Entropy (ApEn), Sample Entropy (SampEn)	Sensitive to data length and noise.
Nonlinear Features	Quantify chaotic behavior and complexity of brain activity.	Lyapunov Exponents, Correlation Dimension	May not be robust in the presence of noise.
Principal Component Analysis (PCA)	PCA reduces the dimensionality of EEG signals by transforming them into uncorrelated principal components, capturing the most significant variance in the data.	Eigenvalue decomposition, Singular Value Decomposition (SVD)	Loss of interpretability of components, requires careful selection of the number of components to retain.
Independent Component Analysis (ICA)	ICA separates mixed EEG signals into independent sources, helping to isolate brain activity from artifacts (e.g., eye blinks, muscle noise).	Fast ICA algorithm, Infomax algorithm	Requires a large number of data samples, may be sensitive to noise, and computationally expensive.
Hilbert-Huang Transform (HHT)	HHT decomposes nonstationary EEG signals into intrinsic mode functions (IMFs), providing a time-frequency representation of brain activity.	Empirical Mode Decomposition (EMD), Hilbert Transform	Computationally intensive, sensitive to noise, and can be difficult to interpret for complex signals.
Recurrence Quantification Analysis (RQA)	RQA quantifies the recurrence behaviour of EEG signals, providing insights into signal regularity, complexity, and transitions in different brain states.	Recurrence plots, Determinism, Laminarity, Entropy	Sensitivity to the embedding parameters, requires large datasets, and can be computationally demanding.

In Time domain analysis, basic statistical features like mean, variance, skewness, and kurtosis are used to describe signal characteristics [24]. Time domain features are often simple to calculate and provide useful insights about signal characteristics but often fail to capture the transient and complex nature of epileptic seizures.

Frequency domain features are useful for analyzing the rhythmic activity of EEG signals [25]. Fast Fourier Transform (FFT) is one of the popular approach to analyse the frequency components of EEG signals [26]. Auto Regressive models focus on modelling the temporal dynamics of EEG signals [27]. Power spectral density, band power, mean frequency, spectral frequency and harmonics are the common frequency domain features extracted. While these techniques work well for stationary data, they fall short in handling the non-stationary signals

Wavelet transform decomposes signals into time-frequency components [28], capturing both the temporal and frequency characteristics of the signal. Wavelet transform has gained prominence due to its ability to simultaneously capture both frequency and temporal dynamics making it effective for detecting non-stationary signals. Wavelet co-efficient, wavelet energy, wavelet band power and power spectrum are the common features extracted. But still challenges exists in handling non-stationary data as the frequency characteristics may vary over time.

Entropy based features measures the randomness, unpredictability and complexity of the signal [29]. These features measure the information content in the EEG signals useful for analyzing brain dynamics under various conditions, such as normal, seizures and other pathological states. They are very essential in identifying irregularities and subtle changes in EEG patterns. The commonly used entropies [30] in EEG signal analysis are Shannon entropy, Approximate entropy, Sample entropy, Renyi entropy, permutation entropy, wavelet entropy and spectral entropy. Each entropy provides unique insights into the signal's complexity serving as a key component in feature extraction and classification tasks.

Nonlinear dynamics has become a powerful approach in measuring irregularities of complex signals [31]. Metrics like fractal dimension, Lyapunov exponents and the Hurst exponent are measured [32]. These features aids in determining the irregular and unpredictable patterns of EEG improving the effectiveness of classification.

Principal Component Analysis (PCA), Independent Component Analysis (ICA), Hilbert-Huang Transform (HHT) and Recurrence Quantification Analysis (RQA) are powerful techniques used in EEG signal analysis. PCA is commonly applied to reduce dimensionality and noise by transforming the high-dimensional EEG data into uncorrelated principal components [33]. ICA is effective in separating neural activity and removing artifacts such as eye blinks and muscle noise [34]. The Hilbert-Huang Transform (HHT) breaks down EEG signals into intrinsic mode functions (IMFs) capturing subtle variations in brain activity, which is useful for seizure detection and adaptive filtering [25]. RQA analyses the recurrence pattern of signals by examining phase space of EEG signal. It provides valuable insights into the dynamics of EEG signals across different brain states, such as seizures and sleep stages [35]. Each of these techniques contributes significantly to EEG signal processing by enabling precise feature extraction, reducing unwanted noise and capturing small changes in brain activity. This makes invaluable contribution in improving the precision and dependability of EEG analysis.

By incorporating temporal, frequency and nonlinear aspects, models can better depict the complexity of brain activity. By integrating this with machine learning and deep learning, the models become even more powerful, allowing for highly accurate predictions.

### Classification approaches

The classification of EEG signals has advanced tremendously over the past few decades contributing to the latest advancement of EEG classification. Various classification methods have been developed, ranging from conventional machine learning methods to cutting-edge deep learning approaches.

i) Traditional Machine Learning Approaches:

Conventional machine learning approaches have been widely used to categorise focal and non-focal epilepsy. These methods typically rely on handcrafted features derived from the EEG signals, including time domain, frequency domain, and nonlinear features. With the advent of machine learning models like Support Vector Machines (SVM), k-Nearest Neighbors (k-NN), Decision tree, Logistic Regression and Naive Bayes classifier became popular for EEG classification.

• Support Vector Machines: SVM is a supervised learning model that identifies the best hyperplane to distinguish classes. SVM has exhibited good performance in classifying EEG data, especially for focal epilepsy detection. However, it faces challenges with the complexity and variability of generalized seizures [36].
• k-Nearest Neighbors: k-NN assigns signals to a class based on the majority vote of their k-nearest neighbors in the feature space. It is simple and can perform well with small datasets. It operates on the principle that similar data points are likely to belong to the same class, based on their proximity in the feature space [37].
• Decision tree: A decision tree is a tree-like structure where each internal node represents a feature, each branch represents a decision rule, and each leaf node represents a class label. The tree is created by continually partitioning the data according to specific criteria. Decision Trees provide a simple, interpretable, and effective classification approach for EEG signals [38].
• Logistic Regression: Logistic Regression is a popular linear classifier that models the association between input features and a binary output class by applying the sigmoid function. It estimates the likelihood of a sample belonging to a specific class. The model outputs a probability between 0 and 1, which is thresholded to make the final class prediction. Logistic Regression is simple, interpretable, and computationally efficient, but it assumes a linear relationship between features and class, which may limit its performance with non-linear EEG data [39].
• Naive Bayes classifier: Naïve Bayes is a probabilistic classification approach based on Bayes’ theorem. It is simple, efficient and capable of handling continuous and categorical data. It assumes that features are independent and computes class probabilities, allocating a sample to the class with the greatest likelihood [40].
• Random Forest: The ensemble method Random Forest incorporates Bagging (Bootstrap Aggregating) to construct various decision trees from randomly selected data subsets. For both classification and regression tasks the multiple outputs of individual trees are combined using majority voting for classification or averaging for regression. The method serves as an effective strategy to minimize overfitting problems especially when dealing with large high-dimensional noisy datasets [41].
• AdaBoost (Adaptive Boosting): It is a powerful ensemble learning technique that constructs a series of classifiers, where each new classifier focuses on improving the errors of the previous classifiers. The final prediction result is obtained from the weighted-balanced combination of individual classification models. Through its iterative process AdaBoost optimizes weak models by improving their accuracy levels. The model is capable of detecting subtle patterns by its weighted mechanics towards the misclassified data thus improving overall prediction accuracy [42].
• Gradient Boosting (GBM): It is another robust method that builds models sequentially, with each model correcting the errors of the previous one by minimizing a loss function. It fits new models to the residual errors of the previous ones. Popular variants like XGBoost and LightGBM are optimized for speed and performance, making them highly effective for large-scale classification tasks [43].

These classifiers, have been extensively applied in EEG signal analysis and rely on manually extracted features, requiring significant preprocessing and expert knowledge to extract the intricate and non-linear patterns embedded in EEG signals. High dimensionality may suffer from overfitting as they are usually prone to noise and artifacts. These limitations of traditional machine learning methods demand the need for more robust techniques like deep learning, as they use multidimensional feature retrieval from the raw signal.

ii) Deep Learning Approaches

Deep learning has revolutionized EEG classification process. The process of feature extraction has become fully automatic using deep learning methods when dealing with complex high-dimensional data sets.

• Convolutional Neural Networks (CNNs): CNNs have the ability to detect spatial patterns which makes them highly effective at analyzing EEG signals. CNNs process input data directly through learning functions which allows them to detect focal seizures effectively [44].
• Recurrent Neural Networks (RNN): RNN's specifically handle sequential data so they become excellent for tasks with temporal dependencies. Standard neural networks differ from RNNs because these networks use structured loops which allow data transmission between consecutive time steps. RNNs possess this ability because of their structural design which lets them identify time-changing patterns including brain signals in EEG data [8].
• Long Short-Term Memory Networks (LSTMs): LSTM is a specialized type of RNN which enables model-based temporal dependency analysis to interpret seizure sequences reliably. The automatic process of learning temporal EEG signal patterns in EEG signals enables LSTMs to achieve improved classification results [45].
• Deep Reinforcement Learning (DRL): DRL functions effectively for EEG classification as it trains the system to choose optimal decision-making approaches through environment interaction, especially for seizures and brain state recognition. The technique works efficiently when decisions must be made one after another since each new decision depends on the preceding EEG observation. DRL executes real-time adaptation which qualifies the technology as an ideal solution for continuous EEG monitoring and helps identify crucial events during database streams [46]..
• Spiking Neural Networks (SNN): SNN's are inspired by the way biological neurons communicate and are designed to closely mimic brain activity. SNN's are still an emerging area of research and has shown promising results for real-time EEG classification, especially in detecting complex patterns like seizures and cognitive states, due to their temporal precision and bio-inspired dynamics [47].

A Comparative Analysis of traditional and deep Learning models is shown in table 4. Deep learning models, have outperformed traditional machine learning methods by eliminating the need for handcrafted features, resulting in better generalization and accuracy.

**iii) Hybrid Models**

**Table 4:** Performance Comparison-Conventional Machine Learning vs. Deep Learning.

Model Type	Examples	Advantages	Disadvantages
Traditional ML Models	Support Vector Machines (SVM), Random Forests, k-Nearest Neighbors (k-NN)	Computationally efficient, easy to interpret, less data-hungry	Requires feature engineering, struggles with complex patterns in data.
Deep Learning Models	Convolutional Neural Networks (CNN), Long Short-Term Memory (LSTM), Autoencoders	Capable of learning complex patterns, no manual feature extraction needed	High computational cost, may lack interpretability.

Hybrid models combine signal processing and machine learning algorithms to achieve superior performance by combining the best aspects of both approaches [48]. These models typically first apply traditional feature extraction methods followed by machine learning classifiers or deep neural networks. Summary of various hybrid model architectures is shown in Table 5.

**Table 5:** Summary of different hybrid model architectures.

Hybrid Model Type	Signal Processing Method	Machine Learning Method	Advantages
Traditional + ML Models	Wavelet Transform, Fourier Transform	SVM, Random Forest, k-NN	Balances feature extraction and classification.
Signal Processing + Deep Learning	Wavelet Transform, ICA	CNN, LSTM, Autoencoders	Captures both spatial and temporal features.

The models showcase flexibility by handling basic and complicated datasets making them appropriate for real-time monitoring as well as advanced clinical usage on a large scale. High accuracy and generalization capabilities characterize them as highly useful tools for managing noisy and incomplete data sources.

### Performance evaluation metrics

A confusion matrix functions as a critical method to evaluate the performance accuracy of classification models [49]. The confusion matrix establishes a direct comparison between model predictions and actual outcomes which provides information about wrong predictions together with their frequency occurrence. The confusion matrix functions as an evaluation instrument to assess model accuracy by providing insights into performance areas that can benefit from improvement when working with either binary or multi-class classification tasks.

Different evaluation metrics enable the assessment of a classification model's operational performance. The model accuracy and reliability get determined through these measurement elements. Table 6 presents an overview of the importance of these metrics for epilepsy classification purposes. The most widespread metrics used are listed below:

• Accuracy: The Accuracy metric determines correct prediction rates (both true positives and true negatives) out of total predictions to reflect model prediction quality.
• Sensitivity (Recall): Sensitivity measures how effectively a model identifies positive instances. It is defined as the proportion of actual positive cases (true positives) that the model correctly identifies as positive.
• Specificity: Specificity measures how effectively a model identifies negative instances. It is defined as the proportion of actual negative cases (true negative) that the model correctly identifies as negative.
• F1-Score: The F1-Score calculates the harmonic average between sensitivity and precision to find equilibrium between recall and precision. The F1-Score delivers maximum value when working with distributions that have unbalanced class ratios.
• Area Under Curve (AUC-ROC): The overall classifier discrimination ability between positive and negative classes is measured using AUC values. AUC represents the total area contained under the ROC curve.

**Table 6:** Description and Relevance of Evaluation Metrics in Epilepsy Classification.

Metric	Description	Importance in Epilepsy Classification
Accuracy	The proportion of correct predictions.	Provides an overall measure of model performance.
Sensitivity (Recall)	True Positive Rate, measures the model's ability to identify focal seizures.	Crucial for minimizing missed focal seizures.
Specificity	True Negative Rate, measures the model's ability to identify non-focal seizures.	Ensures that non-focal seizures are accurately detected.
F1-score	Harmonic mean of precision and recall.	Balances false positives and false negatives.
AUC (Area Under Curve)	Evaluates performance across all thresholds.	Provides a comprehensive measure of model performance.

The combined metrics present a complete framework to study and enhance classification models that detect epilepsy through EEG analysis.

Challenges and Limitations:

While significant advancements have been made, several key challenges remain in the current models:

• **Computational Complexity**: Deep learning models, especially those involving large datasets and real-time analysis, require substantial computational resources. This presents a barrier to the widespread adoption of these models in clinical practice, where high-performance hardware may not always be available [50].
• **Model Interpretability**: Deep learning models, particularly CNNs and LSTMs, are often considered "black-box" models. This lack of interpretability raises concerns in medical settings, where understanding the reasoning behind a diagnosis is crucial [51].
• **Data Scarcity and Generalization**: Medical research faces data issues as the acquisition of high-quality datasets proves hard because of privacy rules along with expensive labeling procedures and inconsistent medical data [52].
• **Real-time Performance**: The primary challenge in real-time categorisation is to get speedy and accurate results with minimal latency. To achieve this, efficient algorithms and optimised hardware are needed [53].

The existing challenges serve as motivation to conduct continuous research on deep learning strategies that enhance model accuracy along with interpretability features for real-time epilepsy categorization systems.

### Future directions

Advancements in machine learning are altering the way the research is carried out on epilepsy using EEG data. Multiple hurdles exist in the field of EEG data analysis due to limitations in accuracy rates and model interpretability and validity regarding clinical setups. The forthcoming section investigates methods to deal with the existing challenges.

i. Advances in Machine Learning

Standard model training methods require large numbers of labeled datasets but acquisition of such data proves challenging in medical settings. The limitations can be addressed by transfer learning and reinforcement learning and multi-task learning techniques.

• Transfer Learning: This approach focuses on leveraging a model that has already been trained on a larger dataset in a different field, and adjusting it to work for domain specific data like epilepsy. Fields such as image classification have benefitted from it, and with increasing developments in deep learning, it is expected to increase classification accuracy for EEG signals from epilepsy patients. This technique utilizes pre-trained models and modifies the details of epilepsy data, which is a complex task. In cases, where there is insufficient labelled data on epilepsy, this approach can greatly enhance classification performance [54].
• Reinforcement Learning (RL): RL is about training models to make decisions based on the feedback received from previous actions taken. This feature makes RL suitable for use in real-time tasks like seizure detection. RL models that interact with real-time EEG data can learn and improve through persistent interaction, which results in the creation of highly precise and adaptive systems for seizure prediction [55].
• Multi-task Learning (MTL): Through MTL modeling techniques several interrelated tasks are learned by a single model which allows information exchange between the tasks. A single model executing MTL can analyze epilepsy classification tasks and forecast seizure times with duration and severity levels which produces better understanding of epilepsy patterns and enhances total prediction quality [56] .

These new techniques enable the improvement of classification model performance when used with minimal data sets which results in better generalization and robustness.

ii. Multimodal Data Integration

A very good prospect is the interpretation of the epilepsy classification based on the multimodal data integration. This integrates EEG with other neuroimaging methods like Magnetoencephalography (MEG), Magnetic Resonance Imaging (MRI), functional Magnetic Resonance Imaging (fMRI) and genetic data. It could also improve the accuracy of the classification and provide a more holistic view of epilepsy.

• EEG and MEG Integration: Integrating EEG and MEG for focal and non-focal epilepsy classification builds on the complementary strengths of both modalities. EEG has high temporal resolution and MEG has better spatial resolution. Hence, the combined signals are more robust and can enhance the accuracy of classification [57].
• EEG and MRI Integration: Integrating EEG with structural MRI can provide important information about the brain regions involved in seizures and thus help to locate the seizure focus. Current research has shown that EEG guided MRI increases the seizure detection rates with high accuracy in focal epilepsy patients. The integration of the two methods can increase the accuracy of diagnosis, therapy and post-treatment surveillance [58].
• EEG and fMRI Integration: Integration of EEG and fMRI is likely to provide good results as fMRI produces real-time brain activity data which reveals neural networks that cause seizures with limited timing precision. Better predictions of seizure initiation and propagation become possible through the combination of fMRI with EEG signals which offers detailed observation of temporal seizure movement through different network nodes [59].
• Genetic Data Integration: The development and progression of epilepsy heavily depend on genetic influences. Combining EEG and genetic data allows the development of new models to recognize various seizure types. Such integration might enable healthcare professionals to deliver better custom-made therapeutic solutions [60].

Healthcare solutions reaches its best potential when health care professionals integrate multimodal approaches to epilepsy type classification.

iii. Personalized Medicine

The development of real-time classification systems for wearable EEG devices represents a significant advancement in epilepsy management. These systems can continuously monitor brain activity, detect seizures as they occur, and initiate immediate interventions, potentially reducing both the frequency and severity of seizures.

• Real-time Monitoring: Wearable EEG devices integrated with real-time classification systems have the potential to improve the quality of life of epilepsy patients by continuous monitoring outside the clinical environments. The system sends automated seizure alert notifications to both patients along with healthcare providers enabling prompt assistance [61] .
• Model Optimization for Wearables: A key challenge in real-time systems is to ensure that the models are both accurate and computationally efficient. While deep learning models are effective, they require heavy resources for deployment on wearables. Three main strategies namely model pruning and knowledge distillation along with quantisation help decrease the architecture size and complexity.
• Low-Latency Systems: Low latency classification is very essential in wearable EEG system. It enables the system to respond immediately to seizures, thus helping to reduce the risk of injury or complications [62].

iv. AI in Clinical Practice

Healthcare faces challenges in model implementation due to necessary requirements of high accuracy and reliability alongside clinical interpretability. As with all high-tech diagnostic aids, there comes the challenge to translate technology from the lab to real life healthcare settings where AI finds its application in clinical practice.

• **Reliability and Validation**: AI models need to pass through extensive clinical trials both for proving reliability and safety. Standard healthcare practices will accept AI systems through validation processes which help establish system trustworthiness [63]. The systems possess continuous learning abilities which let them adjust to new data throughout the monitoring process and gradually enhance their performance with increased clinical data accumulation.
• **Model Interpretability**: Healthcare professionals need interpretability features for deploying artificial intelligence models successfully in medical environments. XAI plays a critical role to enable healthcare professionals understand AI decision mechanisms thus promoting their acceptance of AI technologies at medical sites and instilling confidence in their decision-making abilities [64].
• **Clinical Workflow Integration**: AI systems become practical for clinical usage when they merge seamlessly with current medical work processes [65]. The system must provide simplified user interface design in addition to real-time clinical assistance resulting in results which the professionals can understand and perform actions with ease. The purpose of these models should be to support clinical expertise by providing valuable assistance that adds worth while avoiding excessive medical complexity for health professionals.

The successful use of artificial intelligence in clinical settings brings revolutionary changes to epilepsy care through immediate and accurate medical treatments for patients.

## Conclusion

Differentiating focal and non-focal epileptic patterns using EEG signals has advanced significantly. Transitioning from classical signal processing methods to advanced machine learning and deep learning frameworks. These developments have not only enhanced classification accuracy but also contributed to the broader goals of personalized medicine, real-time monitoring, and proactive seizure management. This section provides a holistic summary highlighting the critical insights, explores the potential clinical impacts of these advancements, and proposes a roadmap for future research directions.

The technology has witnessed a significant shift from conventional signal analyzing methods such as Fourier Transform, Autoregressive models and Wavelet Transform to advanced machine learning and deep learning approaches. While the traditional methods laid the foundation, they were limited in capturing the non-stationary nature of seizure signals and often relied on traditional feature extraction, which was computationally expensive and time-demanding. The advent of machine learning techniques, including Support Vector Machines (SVM) and k-Nearest Neighbors (k-NN), improved performance by automating feature learning, but they still struggled with handling complex seizure patterns and large datasets.

The introduction of deep learning models, notably CNN, LSTM and RNN, marked a major breakthrough. These models demonstrated superior abilities to automatically extract features from raw EEG data, leading to enhanced classification accuracy and improved generalization across diverse patient populations. CNNs are particularly effective in spatial analysis, such as detecting focal seizures, while LSTMs excel at capturing temporal dynamics, especially for non-focal seizures, were activity spreads across multiple brain regions. However, challenges remain in data scarcity, computational cost, and model interpretability, which hinder the seamless integration of deep learning models into clinical practice.

The technological improvements create significant implications for clinical practice which leads to patient care improvements through better precision and rapid personalized treatment of epilepsy. Key benefits include:

• Earlier Seizure Detection: Better classification accuracy enables doctors to detect seizures quickly thus reducing the dangers of seizure durations beyond control [66]. Early seizure recognition enables the rapid initiation of protective measures which consequently protects patients from potential dangers and severe consequences. Automated systems perform essential notifications to clinicians or caregivers who use this information to initiate prompt actions that enhance patient safety.
• Personalized Treatment Plans: The treatments can be made personalised by using machine and deep learning techniques by analysing individual EEG patterns. These models enable personalised treatment based on factors like seizure type and brain activity. Thus, leading to improved results reducing side effects [67].
• Real-Time Seizure Management: Live monitoring through these systems provides data that results in preventative services by sending immediate notifications to patients and their caregivers or medical professionals. The quick delivery of information enables clinicians to decide quickly which could lead to decreased seizure occurrences and severity through immediate intervention [68].

These advances create better healthcare processes which result in personalized treatment solutions for epilepsy patients. The clinical adoption of epilepsy classification through machine learning and deep learning techniques depends on addressing specific fundamental issues.:

• Collaborative Efforts in Dataset Creation: A major challenge in developing robust models is the scarcity of high-quality, labeled datasets [69]. Collaborative initiatives involving academic institutions, hospitals, and industry stakeholders are crucial to create standardized datasets that better reflect the diversity of patient populations, seizure types, and clinical conditions. Addressing class imbalance and data noise is also essential to enhance model effectiveness.
• Model Transparency and Interpretability: One of the primary barriers to clinical adoption of deep learning models is their lack of interpretability. For AI systems to be trusted by clinicians, they must provide transparent and explainable outputs [70]. The implementation of explainable AI (XAI) approaches involving saliency maps and grad-CAM and LIME gives researchers access to model decision-making steps for better understanding of artificial intelligence by human experts.
• Improving Generalization Across Patient Populations: A model needs to function across various patient groups. Variations in EEG signals occur because of different patient characteristics such as age and comorbidity status and seizure origin locations so models need to adapt accordingly [71].
• Deployment in Clinical Settings: AI models require three functional components for successful clinical implementation: they need precise predictions combined with operational efficiency as well as scalability features and a clear ability to generate clinical actions. AI systems must help healthcare providers make decisions as part of their work processes not as a means to replace them [72]. Ensuring that predictions are made in real-time and offer actionable insights will enhance their utility and acceptance in clinical settings.

In conclusion, overcoming these challenges will be critical in accelerating the implementation of AI driven epilepsy classification systems, improving patient outcomes and revolutionizing the management of epilepsy in clinical practice.

## Ethics statements

This work involves does not involve any collection of data.

## CRediT authorship contribution statement

**Rajani Rai B:** Conceptualization, Methodology, Software, Writing – Reviewing and Editing. **Karunakara Rai B:** Supervision, Data analysis. **Mamatha A S:** Visualization, Interpretation of results. **Kavitha Sooda:** Validation, Final review.

## Declaration of competing interest

The authors declare that they have no known competing financial interests or personal relationships that could have appeared to influence the work reported in this paper.
